# Supplementary material for: Association between household food security and infant feeding practices among women with children aged 6–23 months in rural Zambia
Source: PLoS One. 2023 Oct 2;18(10):e0292052. doi: 10.1371/journal.pone.0292052 (PMC10545113; doi:10.1371/journal.pone.0292052)
Supplement: Attachment — Submitted filename: Association between house hold food security and infant feeding practices among women with children aged 6.pdf [file pone.0292052.s002.pdf]

Association between house hold food security and infant feeding practices among women with children aged 6-23 months in rural Zambia.

The paper present overview of household food security and infant feeding practices. The paper is well written and presents interesting findings from population living in rural areas. The sample size is adequate and represents the population adequately.
Following are few suggestions:

Line 9 please change "proved" to proven
Line 19 remove "associated"
Line 55-56 structure of sentence, please change to "List Zambia among the six countries out of 117 countries, suffering from levels of hunger that are classified as alarming.
Line 211 please remove "that"

The study addresses the household food security and infant feeding practices in rural Zambia. While the study has an elegant design there are certain gaps in the study

1. The impact of the total number of children is not taken into account and is neither was discussed as a confounder

2. Position of the 6-23 months old child in the family. It is likely that the food availability was adequate but the number of children older than the index child might impact accessibility and availability of food for the index case

3. Did the investigator ask questions regarding alternative food that the child has access to. What percentage of these children were eating store bought or adult food.

4. How did the investigator measured child eating habit. Despite availability or accessibility of food, child eating behavior is also partially responsible for the overall health of the child. Did investigator provided a food diary to mothers/caregivers to measure the total amount of food intake in day/week or month.
