# Supplementary material for: Association between household food security and infant feeding practices among women with children aged 6–23 months in rural Zambia
Source: PLoS One. 2023 Oct 2;18(10):e0292052. doi: 10.1371/journal.pone.0292052 (PMC10545113; doi:10.1371/journal.pone.0292052)
Supplement: Attachment — Submitted filename: Review_PONE-D-22-27270_R2.pdf [file pone.0292052.s005.pdf]

Manuscript Title: Association between household food security and infant feeding practices among women with children aged 6 – 23 months in rural Zambia

Manuscript Number: PONE-D-22-27270R2

The study topic entitled "Association between household food security and infant feeding practices among women with children aged 6 – 23 months in rural Zambia" is a serious concern in present day as a developing country in Zambia. The manuscript finding and its relating suggestions would be helpful for policy makers, government to improve household food security and infant feeding practices among women with children aged 6 – 23 months in rural Zambia. Authors are suggested a minor corrections in their manuscript which are pointed below—

1. In Introduction part the line "Infant and young child feeding (IYCF) practices directly affect the nutritional status of children 63 under two years of age and, ultimately, impact their survival." if this kind of fact is found in previously recent works then cite more (Line-62).
2. In discussion part "Similar to earlier studies, our study also shows that household food security significantly correlates with MMF, MDD, and MAD [4]" line-419 citation need for Similar to earlier studies for more strengthening the manuscripts.
3. Add Separate section with strength and limitation of the study.
4. Grammatical errors, tenses and syntax should be checked throughout the text.
