# Supplementary material for: Association between household food security and infant feeding practices among women with children aged 6–23 months in rural Zambia
Source: PLoS One. 2023 Oct 2;18(10):e0292052. doi: 10.1371/journal.pone.0292052 (PMC10545113; doi:10.1371/journal.pone.0292052)
Supplement: Attachment — Submitted filename: Response to the Editor_Resubmission.docx [file pone.0292052.s003.docx]

Response to Reviewers and Editor

Editor’s comment:

Your ethics statement should only appear in the Methods section of your manuscript. If your ethics statement is written in any section besides the Methods, please move it to the Methods section and delete it from any other section. Please ensure that your ethics statement is included in your manuscript, as the ethics statement entered into the online submission form will not be published alongside your manuscript.

Response:

We had added a section between the sections for conflict of interest and references, which was called “ethical statement”. **This has been deleted** as the same information was also contained in the “ethical statement” section of the methodology.
