# Supplementary material for: Association between household food security and infant feeding practices among women with children aged 6–23 months in rural Zambia
Source: PLoS One. 2023 Oct 2;18(10):e0292052. doi: 10.1371/journal.pone.0292052 (PMC10545113; doi:10.1371/journal.pone.0292052)
Supplement: Attachment — Submitted filename: Response to Reviewers.docx [file pone.0292052.s004.docx]

RESPONSE TO REVIEWER COMMENTS

Concern #1. In discussion the author made a comment on the inequality in distribution of food among members and its impact on child feeding and MDD. However, this important point was not covered in the text. Statistical analysis taking into account the position of the child or the dietary habits of child was not addressed.

How it has been addressed:

On the question of statistical analysis taking into account the dietary habits of the child, we are unable to do that due to lack of data on the same. This was stated in the previous review and the limitation was acknowledged in the limitation section of the paper. With regard to the issue of the child’s position within the household, this variable cannot be added considering that we collect data on the index child, who happens to be the youngest child in the household. It is very similar to number of under 5 children in the household in that in a household with 5 children, the youngest child will be on position number 5. This similarity introduced multi-collinearity and thus the variable position of child is not included. We also do not discuss the variable in the text for number of children as it was statistically insignificant.

Concern #2. The study was conducted in an area that was supported by world vision Zambia. An important component of the program is educating mothers to improve feeding practises. Was this taken into account during analysis. It is likely that the education mothers are receiving through programs is sufficient for improving feeding practises and not having formal education is therefore not impacting feeding practises. I believe this is an important observation which was not taken into account during analysis.

How it has been addressed:

We have taken advantage of the fact that WVZ did not implement MNCHN programmes across all the intervention areas. To this effect, we added a new variable disaggregating the data between areas with MNCHN interventions and those without so as to assess whether feeding practices are different between these two areas. These changes have been effected in the introduction, results and discussion. However, the inclusion of this variable does not significantly influence the overall results.

Concern #3. Analysis showed that Young children (6-11 months old) were most significantly impacted by maternal feeding practises which makes sense because they cannot fend for themselves. Did author tried to investigate the association between maternal education, feeding practises and impact on child nutrition, I think this is where maternal formal education will have the most significant impact.

How it has been addressed:

The authors appreciate that investigating the association between maternal education, feeding practices and impact on child nutrition is very important. However, the focus of the paper was on association between household food security and infant feeding practices among women with children aged 6 – 23 months. While the impact of education on child feeding practices is investigated and discussed, we do not look at the three-way interaction between maternal education, feeding practices and child nutrition outcomes due to data limitations. However, this limitation has been acknowledged in the paper and included as a potential area for future studies.
