# Supplementary material for: Association between household food security and infant feeding practices among women with children aged 6–23 months in rural Zambia
Source: PLoS One. 2023 Oct 2;18(10):e0292052. doi: 10.1371/journal.pone.0292052 (PMC10545113; doi:10.1371/journal.pone.0292052)
Supplement: Attachment — Submitted filename: Response to reviewers comments 11.09.2023.docx [file pone.0292052.s006.docx]

Response to reviewer’s comments

1. In Introduction part the line ―Infant and young child feeding (IYCF) practices directly affect the nutritional status of children 63 under two years of age and, ultimately, impact their survival. If this kind of fact is found in previously recent works, then cite more (Line-62).
   - Response
     - We have cited previous works showing the association between IYCF and child nutrition status as well as literature showing association between IYCF and child survival. We have also included statistics showing the proportion of child deaths arising from malnutrition. The reference section has also been adjusted accordingly
2. In discussion part ―Similar to earlier studies, our study also shows that household food security significantly correlates with MMF, MDD, and MAD [4] line-419 citation need for Similar to earlier studies for more strengthening the manuscripts.
   - Response
     - We have cited previous work that found significant association between household food security and child feeding practices. The references have also been adjusted accordingly
3. Add Separate section with strength and limitation of the study.
   - Response
     - The strengths and limitations of the current study were already included within the discussion section of the paper as a paragraph. We have put a section header within the discussion clearly labelled as “strengths and limitations of the study” to make it more visible
4. Grammatical errors, tenses and syntax should be checked throughout the text
   - Response
     - The whole document has been reviewed and grammatical errors, tenses and syntax revised accordingly
